# Exploring the association between *SLC11A1* and *CARD15* gene polymorphisms and tuberculosis susceptibility in Holstein cattle

**DOI:** 10.5194/aab-67-515-2024

**Published:** 2024-10-21

**Authors:** Safa Bejaoui, Nour Elhouda Fehri, Mohamed Amine Ferchichi, Bayrem Jemmali

**Affiliations:** 1 University of Carthage, LR13AGR02, Mateur Higher School of Agriculture, Mateur, Tunisia; 2 University of Carthage, National Agronomic Institute of Tunisia, Research Laboratory of Ecosystems & Aquatic Resources, Tunis, Tunisia; 3 Dept. of Veterinary Medicine and Animal Sciences, University of Milan, Via dell'Università 6, 26900, Lodi, Italy

## Abstract

Bovine tuberculosis (bTB) is a contagious disease that has a socio-economic impact on familial and industrial farms. Genetic selection can improve resistance against bTB. This study aimed to characterize the links between *SLC11A1* and *CARD15* gene polymorphisms and tuberculosis (TB) susceptibility. In total 200 animals were used (50 cases and 150 controls). Polymorphisms in the *SLC11A1* and *CARD15* genes were identified and analyzed with the restriction fragment length polymorphism (PCR-RFLP) method. Our results report the PstI PCR-RFLP marker for the *SLC11A1*-SNP1 site and the StyI PCR-RFLP marker for the CARD51-SNP1 site in Tunisian Holstein cows. Statistical analysis showed a significant association between *SLC11A1*-SNP1, *CARD15*-SNP1 and susceptibility/resistance to TB ( P < 0.05). Two *SLC11A1*-SNP1 genotypes were susceptible to tuberculosis, i.e., the heterozygote CG and the homozygote CC, while one *SLC11A1*-SNP1 genotype, i.e., the GG mutated homozygote, was less susceptible to infection. Concerning the *CARD15* gene, two genotypes are highly sensitive to the incidence of bovine tuberculosis, i.e., AA and AG, while the GG genotype is more beneficial and more tolerant of *Mycobacterium bovis*. *SLC11A1* and *CARD15* are two useful candidate genes associated with tuberculosis, and this information can be used to improve the health status of domestic cattle and humans.

## 1 Introduction

Bovine tuberculosis (bTB) infection is caused by the bacterium *Mycobacterium bovis* (*M. bovis*), a member of the *Mycobacterium tuberculosis* complex, which poses a global threat to livestock and humans (Thoen et al., 2006). Most human cases of tuberculosis are caused by *Mycobacterium tuberculosis*, although small proportions are caused by *M. bovis* from cattle and other domesticated mammals. The world has attempted to eradicate tuberculosis (TB) from their domestic animals to minimize the risk of zoonotic transmission (Borham et al., 2022). In Tunisia, bTB represents a priority notifiable disease, and there has been an established control initiative since the 1980s. The national program to eradicate bovine tuberculosis involves only annual testing, restrictions on animal movements, and the slaughter of contaminated animals (Abid et al., 2019). Research has demonstrated that susceptibility to bTB is affected by various genes linked to the disease and that this susceptibility is hereditary and can be transmitted between populations (Brotherstone et al., 2010; Bermingham et al., 2014; Freitas et al., 2021). Finlay et al. (2012) demonstrated that the onset and severity of bTB infection varies significantly among animals due to genetic differences in their immune systems. Therefore, developing genetic resistance is an effective approach to combat infectious diseases in animals, and breeding animals with greater resistance to *M. bovis* is a viable control strategy. However, two genes, with major effect, have been identified to be associated with animal tuberculosis: the solute-like carrier family 11 A1 (*SLC11A1*), also recognized as NRAMP1 (Natural resistance-associated macrophage protein 1), and nucleotide-binding oligomerization domain-containing protein 2 (NOD2), also known as Caspase Recruitment Domain Protein 15 (*CARD15*) (Wang et al., 2015; Baqir et al., 2016). Since 2006, results have shown that tuberculosis susceptibility or resistance varied regarding the genotype on existing genes (Yen et al., 2006; Pinedo et al., 2009). Holder et al. (2020) and Cheng et al. (2015) have discovered single-nucleotide polymorphisms (SNPs) in the bovine *SLC11A1* gene that are linked to bovine tuberculosis susceptibility. Natural resistance-associated macrophage protein 1 (NRAMP1) is a multi-pass membrane protein that mediates macrophage responses to intracellular parasites during the initial stages of infection (Paixão and Gontijo, 2007; Martínez-Lirola et al., 2008). Additionally, it moderates macrophage activation in both infectious and autoimmune diseases by serving as a transport protein for protons, iron, and other divalent cations (Holder et al., 2020). According to Wang et al. (2015), the *CARD15* gene functions as a pattern recognition receptor (PRR) for bacterial lipopolysaccharide (LPS). LPS triggers the release of inflammatory mediators that detect muramyl dipeptide and activate NF- κ b (nuclear factor kappa B). NF- κ b in turn controls the transcription of tumor necrosis factor α , interferon- γ , interleukin-1 β , and interleukin-12. *CARD15* participates in the cellular immune response and plays a crucial role in generating antibodies against bovine tuberculosis (Allen et al., 2010). SNPs associated with susceptibility and resistance to bovine tuberculosis (bTB) have been identified in a study of Taiwanese and Chinese cattle (Cheng et al., 2015; Liu et al., 2017). Enhancing genetic polymorphism identification proves highly effective in assessing diversity and studying the genetic traits of cattle, facilitating the selection of optimal individuals to fortify herds with heightened immunity. Therefore, in the present study, we examine the link between *SLC11A1* and *CARD15* SNPs and the risk of tuberculosis in Holstein cattle in Tunisia.

## 2 Materials and methods

### 2.1 Ethics approval

Animal welfare was respected, and all procedures in this study were approved by the animal science comity of the National Institute of Agronomy of Tunisia (protocol no. 05/15).

### 2.2 Animals and tuberculosis diagnosis

The experiment was conducted at the experimental farm located in the province of Manouba in northern Tunisia (coordinates: 36.9065544° N, 9.8314227° E). Two hundred Holstein cows were included in the study, with 50 cows classified as cases, testing positive for bTB, and 150 cows as controls, testing negative for bTB. Herds were first examined for bTB, and study animals were selected at random from a list of both cases (infected) and control (non-infected) animals. Used animals have the same habitat and dietary needs; it was assumed that their exposure to *M. bovis* in the environment was uniform. The study's animals were all cows, with ages ranging from 4 to 6 years, and artificial insemination was used for reproduction. The animals were housed in a semi-intensive production system consisting of grass supplemented with a concentrated diet, and they were milked twice daily.

A tuberculin skin test was carried out to identify *M. bovis* as infected or non-infected. In short, animals were given 0.1 mL of *M. bovis* PPD (purified protein derivative) in the caudal fold, and 72 h later the extent of the response was measured. However if the skin thickness was less than or equal to 3 mm , the results were classified negative for tuberculosis. If a cow had a positive result for the single intradermal tuberculin (SIT) test and exhibited symptoms of emaciation, coughing, and dyspnea, it was considered positive for bovine tuberculosis (bTB). Conversely, it was deemed negative only if the same test yielded negative results and the animal displayed no clinical symptoms. This approach integrates both tuberculin test outcomes and clinical symptoms to diagnose bovine tuberculosis in cows.

### 2.3 Blood sampling and bovine genomic DNA preparation

Blood samples collected from the jugular vein were preserved in tubes with 1.5 % ethylene diamine tetra-acetic acid (EDTA). And 5 to 10 mL of peripheral blood was drawn from each animal, kept at - 20 °C , and transported on dry ice to the lab for DNA extraction. Following the manufacturer's recommendations, DNA was extracted from the blood samples using the DNeasy Blood & Tissue Kit (Qiagen, Germantown, MD, USA). Using an ND-1000 spectrophotometer (Nanodrop Technology, Wilmington, DL, USA), the quantities and qualities of DNA were visually confirmed by PCR and restriction fragment length polymorphism (RFLP)-PCR analysis.

### 2.4 PCR and RFLP-PCR analysis

A quantity of 0.2 µL TaKaRa Taq DNA polymerase (5 $U\;µL^{-1}$ , with U representing units) was used to amplify target sequences, in a mix containing 2.5 µL 10 × PCR buffer, 2.5 µL dNTP mixture, 2.5 µL upstream primer (10 $\mathrm{pmol}\;µL^{-1}$ ), 2.5 µL downstream primer (10 $\mathrm{pmol}\;µL^{-1}$ ), 4 µL genomic DNA (10 $\mathrm{pmol}\;µL^{-1}$ ), and 8.3 µL sterile purified water (Tables 1 and 2).

**Table 1:** PCR reagent volumes for the *SLC11A1* and *CARD15* target sequences.

Reagents	Volume ( µL )
dNTP	2.5
Taq	0.2
${\mathrm{MgCl}}_{2}$ (25 mM )	2.5
Buffer	2.5
Primer	2.5
Primer R	2.5
Genomic DNA	4
Pure water	8.3

**Table 2:** Primer sequences of *SLC11A1* and *CARD15* genes

Primers	DNA sequence	Size in base pairs ( bp )	Reference
*SLC11A1*	ATCTCCTTCCTACTGCCCGCACAAACTGTCCCGCGTAG	374	Baqir et al. (2016)
*CARD15*	CAC ATG GGT TCA TCT TTA CTG GGCC TTT TTA TCC CTC TAT CCT CA	377	Wang et al. (2015)

The PCR conditions were as follows. There was 5 min for the first denaturation at 95 °C , followed by 30 cycles of three steps: denaturation at 94 °C for 45 s , annealing at 51–60 °C for 45 s , and extension at 72 °C for 90 s . A final extension at 72 °C for 8 min was performed to have the same size of amplicon. And 2 % agarose gel was used to separate the PCR products. Using 2.5 µL 10 × buffer, 0.3 µL StyI restriction enzyme, 10 µL sterile purified water, 0.2 µL BSA (bovine serum albumin), and 4 µL PCR product (10 $\mathrm{pmol}\;µL^{-1}$ ) in a water bath at 37 °C for 3 to 4 h , an enzyme restriction digestion process was carried out.

### 2.5 Statistical analysis 

SAS software (SAS Institute Inc. SAS^®^ 9.4. 2014) was used to analyze the data. The chi-square $\chi^{2}$ statistical test was used to characterize the link between genetic polymorphisms and on animal's susceptibility to infection. Logistic procedure was used to describe the relationship between genotype and bTB susceptibility. The statistical assessment of the relationship between polymorphisms and bTB was conducted using estimated odds ratios (ORs) and 95 % confidence intervals (CIs). For every SNP, the Hardy–Weinberg equilibrium (HWE) was examined. Differences were deemed statistically significant at p < 0.05.

**Figure 1:**
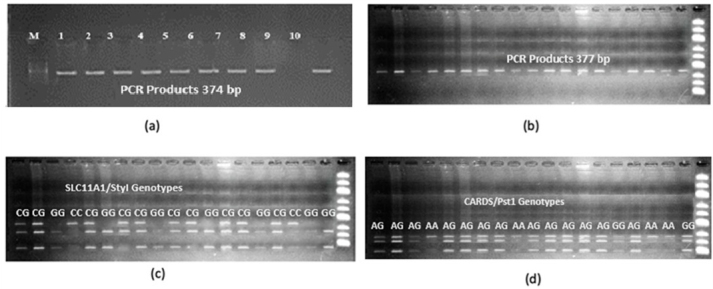
Agarose gel profiles of **(a)** *SLC11A1*-PCR products under UV (marker, *SLC11A1*: 374 bp ), **(b)** *CARD15*-PCR products under UV (marker, *CARD15*: 377 bp ), **(c)** PCR-RFLP gel profile of *SLC11A1*/StyI polymorphism (CC: 374 bp , CG: 374 bp //293 bp //81 bp , GG: 293 bp //81 bp ), and **(d)** PCR-RFLP gel profile of *CARD15*/PstI polymorphism. AA: 273 bp //104 bp , AG: 20 bp //104 bp //253 bp //273 bp , GG: 20 bp //253 bp //104 bp .

## 3 Results and discussion

### 3.1 PCR and PCR-RFLP results

Extracted genomic DNA samples were analyzed separately for the *SLC11A1* and *CARD15* target sequences, and DNA bands were obtained by polymerase chain reaction on a 2 % agarose gel. Figure 1a and b shows PCR profile on agarose gel imaged under UV light. DNA samples obtained from cows were amplified separately for the *SLC11A1* and *CARD15* genes in a PCR device. The polymorphic regions of the studied genes were determined by digestion with the restriction endonuclease enzyme PstI for the first gene and StyI for the second gene. With the *SLC11A1*/StyI polymorphism, the CC genotype is characterized by 374 bp , the CG genotype by 374 bp //293 bp //81 bp , and the GG genotype by 293 bp //81 bp bands. Figure 1c shows an exemplary agarose gel profile of the *SLC11A1*/StyI polymorphism. Theoretical yield tapes have base pair ( bp ) lengths of 273 bp //104 bp for AA, 20 bp //104 bp //253 bp //273 bp for AG, and 20 bp //253 bp //104 bp for GG. Figure 1d presents an exemplary agarose gel profile of the PCR-RFLP result of the *CARD15*/PstI polymorphism.

### 3.2 Alleles and genotype frequencies and genetic equilibrium test results

The genotypes CC, CG, and GG were recognized in the *SLC11A1*/StyI gene regions, and genotypes AG, AA, and GG were recognized in the *CARD15*/PstI gene region. Observed genotypes and allele frequencies are shown in Tables 3 and 4; the Hardy–Weinberg genetic equilibrium test and $\chi^{2}$ test results are shown in Table 5. The allele frequencies of the *SLC11A1*/StyI polymorphism in the studied populations were 0.35 for the C allele and 0.65 for the G allele (Table 3). Although the G allele was the most frequent in the population, the GG genotype in case individuals was 24 %. The CG genotype was 64 % and the CC genotype was 12 % in case animals.

**Table 3:** *SLC11A1* gene, genotype, and allele gene frequencies for case and control cows.

Genotype	*SLC11A1*			
	Size		Frequency (%)	
	Cases	Controls	Cases	Controls
GG	12	94	24	63
CG	32	16	64	10
CC	6	40	12	27
Allele	C		G	
Frequencies (%)	35		65	

**Table 4:** *CARD15* gene, genotype, and allele gene frequencies for case and control cows.

Genotype	*CARD15*			
	Size		Frequency (%)	
	Cases	Controls	Cases	Controls
AA	10	20	20	13.3
AG	37	10	74	6.7
GG	3	120	6	80
Allele	A		G	
Frequencies (%)	27		73	

**Table 5:** Hardy–Weinberg genetic equilibrium test results of gene regions.

Gene	$\chi^{2}$ test value	Probability $>\;\chi^{2}$
*SLC11A1*	62 3117	< 0.0001
*CARD15*	128 1073	< 0.0001

For the *CARD15*/PstI polymorphism in terms of allelic frequency, it found that the A allele had a frequency of 0.27 and the G allele had a frequency of 0.73 (Table 4). The G allele was notably prevalent in the studied herds, and the GG genotype was more common in control animals (80 %) compared to case animals (6 %). Conversely, the AG and AA genotypes were more frequent in case animals, at 47 % and 20 %, respectively, compared to 6.7 % and 13.3 % in controls. The $\chi^{2}$ test was employed to characterize the link between genetic polymorphisms and an animal's susceptibility to infection. The heterogeneity of odds ratios (ORs) for susceptibility to bTB infection was evaluated to investigate possible attachment between allele or genotype frequencies and infection status of cattle. To estimate the risk of disease in animals, ORs and 95 % confidence intervals (CIs) were calculated. Statistical significance was set at 5 % ( p < 0.05).

**Table 6:** Maximum probability estimates of regression coefficients.

Factors	Estimate β	Standard error	OR (95 % confidence interval)
*SLC11A1*: rs109453173 C > G			
CC	0.7963	0.1772	2.217
CG	- 1.3957	0.1785	0.248
GG	–	–	1
*CARD15*: E10( + 107) A > G			
AA	- 0.6358	0.2142	0.529
AG	- 1.3279	0.2052	0.265
GG	–	–	1

### 3.3 Effects of polymorphisms on bTB risk

The studied populations were in HWE for the SNP for the *SLC11A1* gene. The $\chi^{2}$ probabilities indicated that the rs109453173 C > G SNP of the *SLC11A1* gene was significantly linked with bTB infection in studied animals in Tunisia (Table 5). This association was also observed by Kadarmideen et al. (2011) in African cows. Liu et al. (2017) reported that *SLC11A1* was significantly associated with susceptibility/resistance to tuberculosis in Chinese Holstein cattle ( P < 0.01). On the other hand, the genotype observed at the studied locus, rs109453173 C > G, has no significance for susceptibility to tuberculin reaction in cattle in India according to Baqir et al. (2016). In this study, the *SLC11A1* and *CARD15* genes have a significant impact on the prevalence of bovine tuberculosis and the animal's immune response to *Mycobacterium bovis*. The resistance capacity of cattle with the CC/*SLC11A1* genotype was 21 % higher than that of the GG/*SLC11A1* genotype. The susceptibility of cattle with the CG/*SLC11A1*1 genotype was 24 % higher than that of the GG genotype. In addition, the C allele was associated with an increased risk of developing bTB, whereas the G allele was linked to a greater tolerance of the infection. It is therefore tempting to suppose that cattle carrying the alternative G allele show increased resistance to bovine tuberculosis due to increased expression of *SLC11A1* in their macrophages, which would be supported by results recently published in Chinese cattle.

In this same study, the authors use logistic regression to characterize the link between *SLC11A1*-SNPs and TB susceptibility. Observed results show a strong association with *SLC11A1*-SNP1, *SLC11A1*-SNP3, and *SLC11A1*-SNP5 with bovine tuberculosis susceptibility/resistance ( p < 0.05). Based on odds ratios and 95 % confidence intervals, they demonstrated that the rate of the *SLC11A1*-SNP1 CC genotype in the control population (32.3 %) was considerably greater than in the case population (16.2 %), suggesting that the CC genotype could provide significant resistance to tuberculosis compared with the TT and TC genotypes. The *SLC11A1* gene is found in the endosomal and phagosomal membranes of macrophages and monocytes and plays a central role in inhibiting *Mycobacterium bovis* proliferation through its involvement in phagosome acidification. In addition, *SLC11A1* regulates levels of divalent cations and nitric oxide, which confer antimicrobial effects and contribute to protective immune responses. This confirms the functional association with the *SLC11A1* gene with an increased level of protection against mycobacteria. Recent evidence has shown substantial hereditary variation in the susceptibility of individual Holstein cattle to bovine tuberculosis, considering the importance of genetic polymorphism in the ability to control the frequency and severity of bovine tuberculosis (Cheng et al., 2015). This could support the hypothesis that *SLC11A1* genetic polymorphisms in bovines are maintained in cow populations by a process of balanced selection, a suggestion that has also been described for the human *SLC11A1* gene.

The population was in HWE for the SNP on the *CARD15* gene (Table 5). The $\chi^{2}$ probabilities revealed that the E10( + 105) A > G SNP of the *CARD15* gene was significantly linked with bovine tuberculosis infection in Tunisian cattle. The resistance of animals with the AA genotype was 52 % higher compared to the GG genotype (Table 6). The susceptibility of animals with the AG genotype was 26 % lower than the GG genotype. The AG or AA genotypes of SNP E10( + 107) had a lower relative risk than GG. The C allele is associated with greater resistance to bovine tuberculosis, while the G allele is associated with greater susceptibility, and its presence makes the population more vulnerable and increases the risk of contracting the disease. Wang et al. (2015) demonstrated that polymorphic alleles of the *CARD15* gene are associated with resistance to bovine tuberculosis but showed that the GG genotype was more resistant than the AG and AA genotypes, which is completely different from our results. The CARD15 protein is involved in the cellular immune response and plays an important role in producing antibodies. A mutation in the *CARD15* gene that leads to changes in the structure of the protein may be a risk factor for tuberculosis (Azad et al., 2012). Wang et al. (2015) performed a meta-analysis to assess the association between *CARD15* polymorphisms and TB risk. They found that one SNP was a protective factor against TB, while polymorphisms in two other SNPs were not associated with TB susceptibility. In their study, they examined the associations between genetic variations in the *CARD15* gene and the incidence of bovine tuberculosis in a Chinese cattle herd. They examined six SNPs, including one similar to the one analyzed in our study (E10( + 107)). According to this study, E10( + 107) was identified as the only SNP with three genotypes, namely, A/A, G/G, and A/G. Consequently, they proceeded to analyze E10( + 107) using various models. They found similar results to our study, with a higher G/G distribution in controls (93.9 %) than in cases (55.3 %), while the A/G and A/A distribution was higher in cases (41.8 % and 2.9 %, respectively) than in controls (5.1 % and 1 %, respectively). As we found in our analysis, they concluded that A/G or A/A genotypes of SNP E10( + 107) had a higher relative risk than G/G.

## 4 Conclusions

The *CARD15* and *SLC11A1* genes represent our first step in exploring the influence of genetic factors on tuberculosis susceptibility of cow herds. We identified an SNP in the *SLC11A1* gene and an SNP in the *CARD15* gene using two primers. Our results suggest that genetic variation in the *SLC11A1* (C > G) gene (resulting in a cytosine to threonine amino acid change) and genetic variation in the *CARD15* (A/G) gene may contribute to the onset and development of bTB, supporting the hypothesis that polymorphisms in this gene are associated with the risk of bTB in Holstein cattle. Bovine TB is a complicated disease that is probably influenced by polymorphisms in many genes, particularly those related to the immune system and host–pathogen interactions. To confirm these results and to characterize the biological mechanism underlying *CARD15* and *SLC11A1*-mediated susceptibility to bovine tuberculosis, gene expression and association studies in larger populations are needed.

## Data Availability

The datasets used in this study are available upon request from the author.
